# Case Report: Multifocal biphasic squamoid alveolar renal cell carcinoma

**DOI:** 10.12688/f1000research.8451.2

**Published:** 2016-05-24

**Authors:** Jose Ignacio Lopez

**Affiliations:** 1 Department of Pathology, Cruces University Hospital, Biocruces Health Research Institute, University of the Basque Country (UPV/EHU), Barakaldo, Spain

**Keywords:** Kidney, biphasic squamoid alveolar renal cell carcinoma, papillary renal cell carcinoma, immunohistochemistry

## Abstract

A multifocal biphasic squamoid alveolar renal cell carcinoma in a 68-year-old man is reported. Four different peripheral tumor nodules were identified on gross examination. A fifth central tumor corresponded to a conventional clear cell renal cell carcinoma. Biphasic squamoid alveolar renal cell carcinoma is a rare tumor that has been very recently characterized as a distinct histotype within the spectrum of papillary renal cell carcinoma. Immunostaining with cyclin D1 seems to be specific of this tumor subtype. This is the first reported case with multifocal presentation.

## Introduction

The so-called biphasic squamoid alveolar renal cell carcinoma (BSARCC) was described for the first time in 2012 by Petersson *et al.* ^1^ and has been very recently revisited and fully characterized by Hes *et al.* ^2^. Histological, immunohistochemical, comparative genomic hybridization and fluorescence *in situ* hybridization analyses have revealed that BSARCC is a renal neoplasm closely related to papillary renal cell carcinoma (PRCC) ^2^.

 The present paper describes a new BSARCC with multifocal presentation that was associated with a conventional clear cell renal cell carcinoma (CCRCC). To note, multifocality has not been reported in BSARCC so far.

## Case report

**Figure 1.**
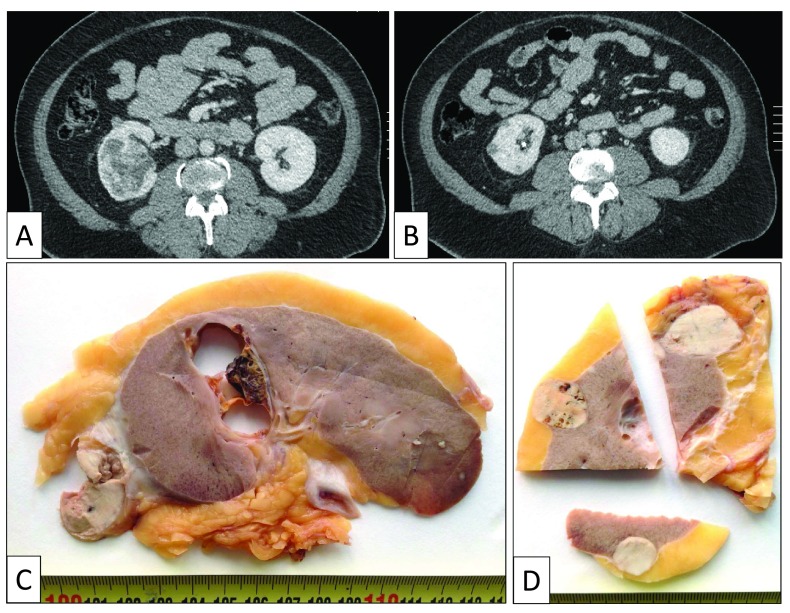
CT scans show multiple tumors in the right kidney ( **A** and **B**). Gross examination displays a yellowish central tumor with solid-cystic areas corresponding to a clear cell renal cell carcinoma ( **C**) and four peripheral whitish tumors and several intrarenal micronodules corresponding to biphasic squamoid alveolar renal cell carcinomas ( **C** and **D**).

A 68-year-old man presented with transient hematuria. CT scan revealed multiple tumors on his right kidney, four of them being located at the periphery ( Figure 1). Radical nephrectomy was performed. Post-surgery period did not show any clinical complication. The patient is asymptomatic and free of disease at the last contact, 6 months after diagnosis.

On gross examination up to five tumors and several small intrarenal micronodules were discovered ( Figure 1). Four tumors were subcapsular and showed a whitish homogeneous cut surface, measuring between 1 and 3 cm in diameter. The fifth tumor was centrally located, presented mixed solid and cystic areas with a yellowish cut surface and measured 4.5 cm in diameter. 

**Figure 2.**
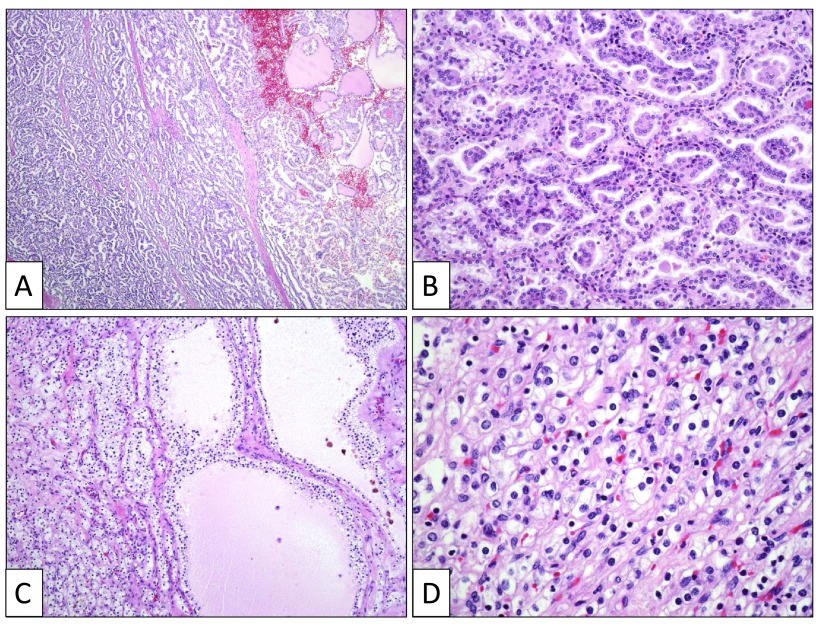
Panoramic view of both tumors, Biphasic squamoid alveolar renal cell carcinoma (BSARCC) ( **A** and **B**) and conventional renal cell carcinoma (CCRCC) ( **C** and **D**). BSARCC displayed some areas of type1 papillary renal cell carcinoma ( **A**, right side) and presented the typical alveolar structures filled with large cells ( **A**, left side and **B**). CCRCC showed solid and cystic areas composed of nests low-grade cells with clear cytoplasm ( **C** and **D**).

**Figure 3.**
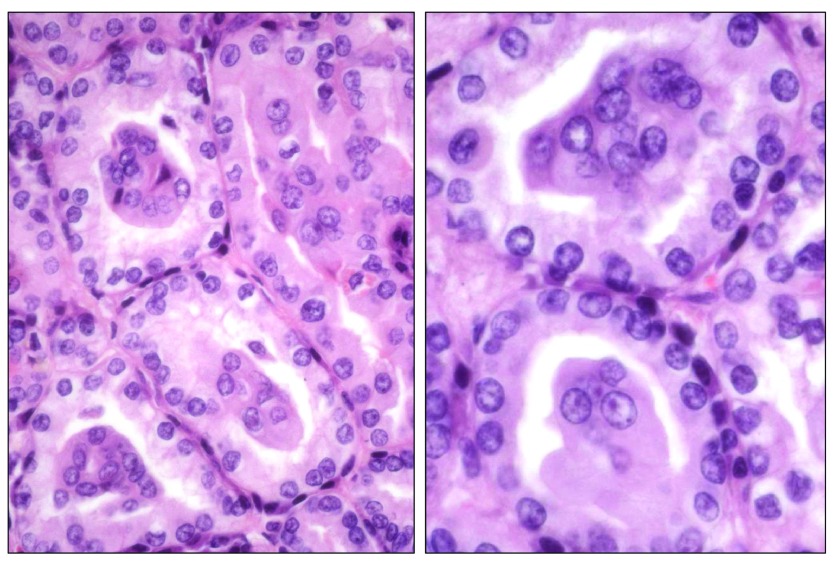
Microscopic detail of the alveolar structures containing small groups and single large squamoid cells ( **A** and **B**).

Histologically, the yellowish central tumor was a conventional organ-confined CCRCC grade 1 (ISUP 2013) ^3^ ( Figure 2). On low-power view, all the whitish peripheral tumors and the micronodules displayed a similar histology consisting in areas reminiscent to glomerular-like structures ( Figure 2 and Figure 3) alternating with others typical of type 1 PRCC. On high magnification, these structures were composed of a single row of small cells with scant cytoplasm displaying an alveolar disposition. The alveoli were filled with cell groups with large cytoplasm and squamoid appearance ( Figure 3). True squamous cell differentiation, however, was not observed. Mitosis and necrosis were not seen.

**Figure 4.**
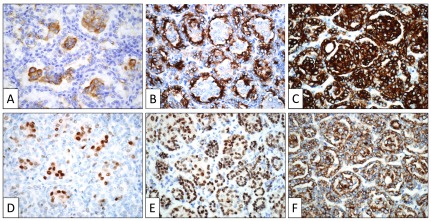
Immunohistochemical study with 34βE12 ( **A**), RCC marker ( **B**), CK7 ( **C**), cyclin D1 ( **D**), PAX-8 ( **E**) and AMACR ( **F**). Noteworthy, 34βE12 and cyclin D1 selectively immunostain the central squamoid cell groups and, conversely, RCC marker does it only in peripheral alveolar cells. CK7, PAX-8 and AMACR immunostain both cell types.

By immunohistochemistry ( Figure 4), the tumor was positive with CK7, vimentin, PAX-8, racemase, RCC marker, AE1/AE3, 34βE12, carbonic anhydrase IX, CD10, and cyclin D1(SP4-R clone, Ventana, USA). Immunostaining pattern was distinct depending on the cell type. For instance, cyclin D1 and 34βE12 immunostained selectively the squamoid cells whilst RCC marker and carbonic anhydrase IX did it only in small alveoli-forming cells. The rest of the antibodies immunostained both cell types. The tumor was negative with p63 and CK20.

## Discussion

BSARCC is a recently recognized variant of renal carcinoma ^1, 2^. Its pathological diagnosis can be suggested on hematoxylin-eosin slides and is based on the recognition of two different cell types arranged in a distinct architecture. Small groups of large cells with abundant cytoplasm and squamoid appearance are surrounded by small cells with scant cytoplasm forming alveolar-like structures. This distinct growth pattern can be more or less evident in different tumor areas and, same as happens in the case here presented, can be combined with areas of conventional PRCC ^2^. The combination of BSARCC and PRCC histologies in almost half of the previously published cases favors the inclusion of this tumor within the broad spectrum of PRCC ^2^. No association of BSARCC with CCRCC, as in the case here presented, has been reported so far.

Morphological diagnostic features of BSARCC can be supported by immunohistochemistry and, if necessary, by genetics. All BSARCC reported to date are positive with cytokeratin 7, epithelial membrane antigen, vimentin and cyclin D1. To note, cyclin D1 shows a selective immunostaining restricted to the central squamoid cell groups. This distinct cyclin D1 distribution seems to be specific of this tumor and may be of help in its recognition. This marker, however, can also immunostain other renal cell neoplasms, as recently reported ^4– 6^. Molecular-genetic data show gains of chromosomes 7 and 17, thus linking BSARCC to PRCC.

## Consent

Written informed consent was obtained from the patient for publication of this case report and any accompanying images and/or other details that could potentially reveal the patient’s identity.

## References

[1] Petersson F Bulimbasic S Hes O: Biphasic alveolosquamoid renal carcinoma: a histomorphological, immunohistochemical, molecular genetic, and ultrastructural study of a distinctive morphologic variant of renal cell carcinoma. *Ann Diagn Pathol.* 2012;16(6):459–469. 10.1016/j.anndiagpath.2012.08.007 23036259

[2] Hes O Condom Mundo E Peckova K: Biphasic squamoid alveolar renal cell carcinoma: A distinctive subtype of papillary renal cell carcinoma? *Am J Surg Pathol.* 2016;40(5):664–675. 10.1097/PAS.0000000000000639 26999503

[3] Delahunt B Cheville JC Martignoni G: The International Society of Urological Pathology (ISUP) grading system for renal cell carcinoma and other prognostic parameters. *Am J Surg Pathol.* 2013;37(10):1490–1504. 10.1097/PAS.0b013e318299f0fb 24025520

[4] Leroy X Camparo P Gnemmi V: Clear cell papillary renal cell carcinoma is an indolent and low-grade neoplasm with overexpression of cyclin-D1. *Histopathology.* 2014;64(7):1032–1036. 10.1111/his.12359 24382138

[5] Lima MS Pereira RA Costa RS: The prognostic value of cyclin D1 in renal cell carcinoma. *Int Urol Nephrol.* 2014;46(5):905–913. 10.1007/s11255-013-0602-0 24242739

[6] Zhao W Tian B Wu C: DOG1, cyclin D1, CK7, CD117 and vimentin are useful immunohistochemical markers in distinguishing chromophobe renal cell carcinoma from clear cell renal cell carcinoma and renal oncocytoma. *Pathol Res Pract.* 2015;211(4):303–307. 10.1016/j.prp.2014.12.014 25596994

