# Supplementary material for: Case Report: Multifocal biphasic squamoid alveolar renal cell carcinoma
Source: F1000Res. 2016 May 24;5:607. Originally published 2016 Apr 8. [Version 2] doi: 10.12688/f1000research.8451.2 (PMC4850880; doi:10.12688/f1000research.8451.2)
Supplement: Peer review history

# Peer review: Referee response for version 2

**DOI:** 10.5256/f1000research.9388.r14154

**Published:** 2016-06-03

**Authors:** Guido Martignoni, Anna Calió

**Affiliations:** 1 Department of Pathology, University Hospital, Ospedale Policlinico, AOUI Verona, Verona, Italy; 2 University of Verona, Verona, Italy

The author has sufficiently addressed our concerns.

We have read this submission. We believe that we have an appropriate level of expertise to confirm that it is of an acceptable scientific standard.

# Peer review: Referee response for version 2

**DOI:** 10.5256/f1000research.9388.r13949

**Published:** 2016-05-25

**Authors:** Claudio Doglioni

**Affiliations:** 1 Anatomy and Histopathology Department, IRCCS (Scientific Institute of Hospitalization and Care), San Raffaele Scientific Institute, Vita-Salute San Raffaele University, Milan, Italy

I have read this submission. I believe that I have an appropriate level of expertise to confirm that it is of an acceptable scientific standard.

# Peer review: Referee response for version 1

**DOI:** 10.5256/f1000research.9099.r13306

**Published:** 2016-04-28

**Authors:** Claudio Doglioni

**Affiliations:** 1 Anatomy and Histopathology Department, IRCCS (Scientific Institute of Hospitalization and Care), San Raffaele Scientific Institute, Vita-Salute San Raffaele University, Milan, Italy

The Author describes an unusual multifocal presentation of Biphasic squanoid alveolar renal carcinoma associated with a conventional Clear Cell Carcinoma. BSARCC is a recently described rare entity with a peculiar squamoid differentiation characterized by cyclin D1 immunoreactivity; Cyclin D1 per se is not limited to this type of renal tumor, but its pattern of reactivity seems specific for BSARCC. I suggest, considering some, although limited, similarity to squamoid morules in other epithelial tumors (colon, endometrium) to perform also immunostaining for beta-catenin.

I have read this submission. I believe that I have an appropriate level of expertise to confirm that it is of an acceptable scientific standard.

# Peer review: Referee response for version 1

**DOI:** 10.5256/f1000research.9099.r13402

**Published:** 2016-04-28

**Authors:** Jose Luis Rodriguez-Peralto

**Affiliations:** 1 Department of Pathology, Hospital 12 de Octubre, Complutense University of Madrid, Madrid, Spain

The authors report for the first time a multifocal variant of the recently described squamoid alveolar renal cell carcinoma, a distinct microscopic tumor in the spectrum of papillary renal cell carcinoma.. This is the first reported case on this location. The paper is original, elegant and well written, and the quality of figures nicely reflect the morphologic, and immunohistochemical aspects of this infrequent neoplasm. The references are acceptable and recent. Thus, I consider this paper acceptable in the current version, but some changes may improve the content of the manuscript:

- Since Cyclin D1 has also been reported in other variant of renal cell carcinoma, I recommend to the author to delete in the discussion the sentence Cyclin D1 immunohistochemistry is specific of this tumor. Moreover, I also included a sentence emphasizing that although Cyclin D1 is characteristic of BSARCC, it may be rarely observed in other variants of RCC.
- I considered that there are many immunohistochemical pictures of Cyclin D1, I would change figure C by a new H&E of the conventional clear cell renal cell carcinoma

I have read this submission. I believe that I have an appropriate level of expertise to confirm that it is of an acceptable scientific standard.

# Peer review: Referee response for version 1

**DOI:** 10.5256/f1000research.9099.r13307

**Published:** 2016-04-20

**Authors:** Guido Martignoni, Anna Calió

**Affiliations:** 1 Department of Pathology, University Hospital, Ospedale Policlinico, AOUI Verona, Verona, Italy; 2 University of Verona, Verona, Italy

The study describes a multifocal biphasic squamoid alveolar renal cell carcinoma in association of clear cell renal cell carcinoma. The Author claims that staining for cyclin D1 is specific for this entity; however, labelling for cyclin D1 has been reported in clear cell renal cell carcinoma, papillary renal cell carcinoma, chromophobe renal cell carcinoma and oncocytoma (1, 2) and more recently in clear cell papillary renal cell carcinoma (3) and SDH-deficient renal cell carcinoma (4). Since the immunoreactivity might depend of the clone used, the Author should supply information regarding the antibody to cyclin D1. It would be also interesting to know whether cyclin D1 is positive in the clear cell renal cell carcinoma. Finally, pictures of clear cell renal carcinoma and more detailed pictures demonstrating the squamoid features of biphasic squamoid alveolar renal cell carcinoma should be added.​

We have read this submission. We believe that we have an appropriate level of expertise to confirm that it is of an acceptable scientific standard, however we have significant reservations, as outlined above.
